# Herpes simplex virus-1 (*HSV*-*1*) infection induces a potent but ineffective IFN-λ production in immune cells of AD and PD patients

**DOI:** 10.1186/s12967-019-2034-9

**Published:** 2019-08-27

**Authors:** Francesca La Rosa, Simone Agostini, Anna Bianchi, Raffaello Nemni, Federica Piancone, Ivana Marventano, Roberta Mancuso, Marina Saresella, Mario Clerici

**Affiliations:** 1 Laboratory of Molecular Medicine and Biotechnology, IRCCS Fondazione Don Carlo Gnocchi, via Capecelatro, 66, 20148 Milan, Italy; 2 Department of Pathophysiology and Transplantation, University of Milan, Milan, Italy

**Keywords:** Alzheimer’s disease, HSV-1, Immune response, IFN-λ, Parkinson’s disease

## Abstract

**Background:**

The sequential activation of immediate early (IE), early (E) and late (L) genes is required to allow productive herpes simplex virus type 1 (HSV-1) infection. Several evidences suggest that, together with inflammation, an immunological response incapable to counteract HSV-1 reactivation plays a role in the pathogenesis of Alzheimer’s (AD) and Parkinson’s (PD) diseases. IFN-lambda (IFN-λ), a cytokine endowed with a robust antiviral activity, contains HSV-1 reactivation. HSV-1-induced IFN-λ, IL-10 and IL-1β as well as the expression of viral IE, E and L genes were analyzed in vitro in peripheral blood mononuclear cells (PBMC) of AD and PD patients as well as of healthy controls (HC).

**Methods:**

PBMC of AD, PD and HC were in vitro infected with one multiplicity of infection (1 MOI) HSV-1. IE, E, and L viral genes transcription as well as IFN-λ, IL-10 and IL-1β production were analyzed.

**Results:**

In HSV-1-infected cells of AD and PD patients compared to HC: (1) transcription of IE (*ICP0*, *ICP27*) genes was reduced whereas that of E (*UL41, UL29*) and L (*UL48, LAT*) genes was increased; (2) IFN-λ mRNA expression was increased. IL-1β was augmented and IL-10 was reduced in unstimulated cells of AD and PD compared to HC; HSV-1 infection significantly increased IL-10 production in HC alone.

**Conclusions:**

Data herein show that a proinflammatory condition is present in AD and PD, in whom attempts to obstacle viral replication via an initial, possibly more potent IFN-λ-mediated control of IE viral genes is unsuccessful.

## Background

Alzheimer’s disease (AD) and Parkinson’s disease (PD) are neurodegenerative disorders characterized by cognitive and behavioral alterations that lead to personality changes and to a decline in cognitive abilities combined with loss of mental function [1, 2]. Both pathologies are characterized by the accumulation in the brain of misfolded proteins [3, 4]: amyloid-beta (Aβ) in AD and alpha-synuclein (α-syn) in PD, as well as by neuroinflammation, which is driven both by microglial cells and by peripheral monocytes which cross the blood brain barrier (BBB) following a gradient of inflammatory cytokines [5]. This is supported both by PET imaging, showing an increased permeability of the BBB, and by the elevated levels of proinflammatory cytokines in the peripheral blood of PD and AD patients [6–8]: the pathogenesis of these pathologies is only scarcely understood but it is known to include genetic [9–11] and environmental factors [12, 13]. Several pathogens are also hypothesized to be involved in the pathogenesis of AD and PD [14–21]; herpes simplex virus type 1 (HSV-1), in particular, is suspected to play a role in these diseases [16, 22–24].

HSV-1 is a double-stranded DNA virus that belongs to the *alphaherpesvirinae* subfamily; HSV-1 infection is widespread as more than 70–90% of the world population are believed to be infected by this virus [25]. After the initial infection of epithelial cells, HSV-1 can spread to central nervous system (CNS) and establish a life-long latent infection in the peripheral nervous system [26]. HSV-1 latent infection is characterized by periodical bursts of reactivation that can be asymptomatic, can cause herpes labialis, or, in rare circumstances, if they take place in the central nervous system, can result in meningitis or encephalitis [27, 28]. HSV-1 reactivations are controlled by the immune response; it is hypothesized that if this equilibrium is lost, excessive HSV-1 replication together with neuroinflammation can become an important factor in the pathogenesis of AD and PD [23, 24, 29, 30].

The production of viral particles observed during viral reactivation depends on the coordinated expression of three classes of viral genes: the immediate early (IE), early (E), and late (L) genes [31]. IE and E proteins along with DNA replication are required for the efficient transcription of L genes [32]. Although HSV-1 uses many strategies of immunoevasion [33], innate and adaptive immune responses are activated to control virus replication and infection: IFN-lambda (λ) in particular plays a key role in containing HSV-1 reactivation [34–39].

To analyze whether HSV-1 replication and innate immune defences differ in AD and PD compared to healthy controls (HC) we used an in vitro model of HSV-1 infection. Results herein indicate that both viral replication and innate immune responses are indeed different when AD and PD cells are compared to those of HC.

## Methods

### Patients and controls

Ten AD and ten PD HSV-1 seropositive patients that fulfilled inclusion criteria for a clinical diagnosis of AD and PD were enrolled from the Rehabilitative Neurology Unit at the Don Carlo Gnocchi Foundation in Milano, Italy. All patients underwent a clinical interview, neurological and neuropsychological examination, laboratory analysis, CT scan or MRI, and other investigations (e.g., EEG, SPET scan, CSF examination, etc.) to exclude reversible causes of dementia. The clinical diagnosis of AD was performed according to the NINCDS-ADRDA work group criteria [40] and the DMS IV–R [41]. Neuropsychological evaluation and psychometric assessment were performed with a Neuropsychological Battery that included: MiniMental State Examination (MMSE), Digit Span Forward and Backward, Logical Memory and Paired Associated Words Tests, Token Test, supra Span Corsi Block Tapping Test, Verbal Fluency Tasks, Raven Colored Matrices, the Rey Complex Figure, Clinical Dementia Rating Scale (CDR) [42, 43].

Diagnosis of PD was based on the Queen Square Brain Bank Criteria [44]. Disease stage has been defined for all the PD according to modified Hoehn and Yahr (HYR) criteria [45]. All but one PD subjects received dopaminergic treatment at the moment of sampling. The study conformed to the ethical principles of the Helsinki Declaration.

Ten sex- and age-matched HSV-1 seropositive healthy controls (HC) as well as six HSV-1 seronegative individuals were also enrolled in the study.

These individuals were selected according to the SENIEUR protocol for immuno-gerontological studies of European Community’s Control Action Program on Aging [46] and were without a family history of dementia or evidence of acute or chronic neurologic diseases at the time of enrollment. The cognitive status of HC was assessed by MMSE (score for inclusion as normal control subjects ≥ 30). Finally, 6 HSV-1-seronegative individuals (3AD, 3HC) were also enrolled in the study.

Informed consent was obtained from all of the blood donors; the ethics committee of the Don Carlo Gnocchi Foundation in Milano, Italy approved the study.

### Analysis HSV-1 seropositivity

Sera that had been banked at − 80 °C were used for serological analysis; serum HSV1-IgG titers were measured using commercially available enzyme immunoassays (ELISA) (IBL International, Hamburg, Germany) in accordance with the manufactures instructions. The assay sensitivity was 98.7%, whereas the specificity was 100%. Subjects with antibody index (AI) > 1.1 were considered seropositive, in accordance with the manufactures instructions.

### Sample preparation

Whole blood was collected in vacutainer tubes containing ethylenediaminetetraacetic acid (EDTA) (Becton–Dickinson & Co., Rutherford, NJ, USA). Peripheral blood mononuclear cells (PBMC) were separated on lympholyte separation medium (Cedarlane, Hornby, Ontario, CA) and washed twice in PBS at 1000 g for 10 min; viable leukocytes were determined using a TC20 Automated Cell Counter (Biorad Hercules, CA, USA).

### Virus preparation

HSV-1 (clinical isolate) was amplified and titred in Vero cells (African Green monkey kidney cells, ATCC CCL81) grown in Dulbecco’s modified Eagle medium (DMEM) containing 2 mM l-glutamine, 1% penicillin (Euroclone, Pero, Milan, Italy) and 10% fetal bovine serum (FBS), and maintained in an incubator with CO_2_ at 5% and 37 °C. For virus amplification, monolayers of Vero cells were infected with 0.01 multiplicity of infection (MOI)(1 plaque formation units (PFU) per 100 cells) of HSV-1 and incubated at 37 °C with CO_2_ at 5%. Seventy-two hours later infected cells were submitted to freeze–thaw and the viral suspension was aliquoted and stored at − 80 °C. Virus titation was performed in duplicate using six wells plates. Virus was submitted to limiting dilution (10^−1^ to 10^−6^) and 400 µL of each dilution were inoculated on Vero cells and incubated at 37 °C in 5% CO2 for 1 h and 30 min. The inoculum was then removed and 3 mL of DMEM plus 2% low-melting point agarose were added to each well. After 72 h, viral titers were calculated using the plaque-forming unit/ml (PFU/mL) method [47].

### Virus-infection

PBMC were cultured overnight in RPMI 1640 supplemented with 10% human serum, 2 mM l-glutamine, and 1% penicillin (medium)(Euroclone). 1.3/10^6^ cells/well plated in 96-wells plates were incubated for 1 h at 37 °C in 5% CO_2_ with serum-free RPMI alone (Medium) or with 1 MOI HSV-1 [36, 48].

### Cells culture

After infection, PBMC were washed with phosphate-buffered saline (PBS) and were cultured in 6-wells plate with RPMI 1640 supplemented with 2 mM l-glutamine, 1% penicillin and 2% human serum. One, 4, and 6 h (see Additional file 1) post infection (p.i.) cells were collected for RNA extraction; 24 h p.i. supernatants were recovered used for cytokines quantification.

### HSV-1 DNA extraction

HSV-1 DNA was extracted from supernatants of culture cells using the spin-columns technique (Nucleospin Tissue, Macherey–Nagel, Duren, Germany) according to the manufacturer instructions. DNA concentration was measured using a spectrophotometer (OD: 260 nm).

### *ApoE* and *IFN*-λ genotyping

Genomic DNA was isolated from whole blood by phenol–chloroform extraction. Customer-design Taqman probes for the 112 and 158 codons were used to determine the genotype of *Apolipoprotein E* gene (*ApoE*) [49].

Single nucleotide polymorphism (SNP) *rs12979860 IFN*-λ was typed as previously described [50].

### RNA-extraction

Total mRNA was extracted from unstimulated PBMC as well as from PBMC 1, 4 and 6 h after HSV-1 infection using the RNA easy Mini extraction kit (Qiagen, Hilden, Germany) and was eluted in RNAse-Free water. Total RNA concentration was determined by spectrophotometer (OD: 260 nm). Purity was determined as the 260 nm/280 nm OD ratio with expected values between 1.8 and 2.0. RNA was treated with TURBO DNA free DNAse (Ambion INC, Austin, TX, USA) and retro transcribed with High-capacity cDNA Reverse Transcription Kit (Applied Biosystems, Foster City, CA, USA), as specified by manufacturers. cDNA samples were stored at – 20 ^°^C until use.

### Analysis of gene expression by reverse transcription-polymerase chain reaction (RT-PCR)

Quantitative RT-PCR was performed using the RT_2_ SYBR Green qPCR mastermix (Qiagen, Hilden, Germany); glyceraldehyde 3-phosphate dehydrogenase (GAPDH), IFN-λ expression was analyzed 4 h p.i. Primers (Qiagen, Hilden, Germany) were cDNA specific; after calculation of 2^ΔΔCt^ (where Ct is the cycle threshold) between the target gene and GAPDH housekeeping mRNA, results were expressed as ratios between stimulated and unstimulated (medium) cells (n-Fold). Experiments were individually run on each one of the individuals included in the study.

qPCR using TaqMan universal master mix (Life Technologies, Carlsbad, CA, US) was used to quantify exogenous HSV-1 copies and viral genes. The HSV-1 DNA polymerase gene (UL30) was amplified to estimate the amount of viral DNA in each supernatant (copies/ml). Immediate early *RL2/ICP0* and *UL54/ICP27* (RNA extracted 1 h p.i.), early *UL29/ICP8* and *UL41* (RNA extracted 4 h p.i.), and late *UL48/VP16* and *LAT* genes (RNA extracted 6 h p.i.) were amplified from cDNA. The *YWHAZ* gene (ID: Hs03044281_g1, Life Technologies) was used as reference. qPCR amplifications were performed by Biorad CFX Real-Time PCR instrument (Biorad, Hercules, California, USA); all experiments were performed in triplicate. For primers and probe sequences, see Table 1; the primers used for HSV-1 genes were selected on the base of previous published works [51–55], but a cross-reactivity with other endogenous viruses, most specifically with the closely related HSV-2, cannot be excluded.

**Table 1:** Primers and probes used for quantification of HSV-1 viral genes

Gene name	Protein name	Primer/probe	Sequence	References
*RL2*	ICP0	Primer F	5′-AACTCGTGGGTGCTGATTGAC-3′	[51]
		Primer R	5′-CAGGTCTCGGTCGCAGGGAAAC-3′	
		Probe	5′FAM-AGCCCGCCCCGGATGTCTGGG-TAMRA 3′	
*UL29*	ICP8	Primer F	5′-CACCAGGTTGCGCATCAG-3′	[53]
		PRIMER R	5′-CTGCATACGGTGGTGAACAAC-3′	
		Probe	5′FAM-ACCTCGCGGTCCACG-TAMRA-3′	
*UL54*	ICP27	Primer F	5′-CGCCAAGAAAATTTCATCGAG-3′	[49]
		Primer R	5′-ACATCTTGCACCACGCCAG-3′	
		Probe	5′FAM-CTGGCCTCCGCCGACGAGAC-TAMRA 3′	
*UL30*	HSV pol	Primer F	5′-CATCAGCGACCCGGAGAGGGAC-3′	[50]
		Primer R	5′-GGGCCAGGCGCTTGTTGGTGTA-3′	
		Probe	5′FAM-CCGCCGAACTGAGCAGACACCCGCGC-TAMRA3′	
*UL48*	VP16	Primer F	5′-CCGGGTCCGGGATTTACC-3′	[52]
		Primer R	5′-CTCGAAGTCGGCCATATCCA-3′	
		Probe	5′FAM-CCCCACGACTCCGCC-TAMRA 3′	
*UL41*	UL41	Primer F	5′-GGACATCCGCGACGAAAAC-3′	[52]
		Primer R	5′-AGAAACCTGTCGGCGATATCAG-3′	
		Probe	5′FAM-CTGGCGCGATCTATC-TAMRA 3′	
*LAT*	LAT	Primer F	5′-GCATAGAGAGCCAGGCACAAAA-3′	[52]
		Primer R	5′-ACGTACTCCAAGAAGGCATGTG-3′	
		Probe	5′FAM-TCCCACCCCGCCTGTG-TAMRA 3′	

### ELISA

IL-1β, IFN-lambda (λ) (IL-29) and IL-10 concentration in the supernatants of PBMC that were or were not HSV-1 infected analyzed by ELISA immunoassays according to the manufacturer’s instructions (ThermoFisher, Waltham, MA, USA). A plate reader (Sunrise, Tecan, Mannedorf, Switzerland) was used and optical densities (OD) were determined at 450/620 nm. IFN-lambda (λ) serum concentration was measured using the same immunoassay.

### Statistical analysis

Quantitative data distribution was analyzed by Shapiro–Wilk test. Normally distributed data were summarized as mean and standard deviation (SD), and comparison among groups were analyzed by ANOVA test and Student t test. Not-normally distributed data were summarized as median and interquartile range (IQR: 25th and 75th percentile), and comparisons were analyzed by Kruskal–Wallis test and Mann–Whitney U test, as appropriate. Data analysis was performed using the MedCalc statistical package (MedCalc Software bvba, Mariakerke Belgium). p-values < 0.05 were considered statistically significant.

## Results

### Demographic and clinical characterization

The demographic and clinical characterization of the individuals enrolled in the study is shown in Table 2. Gender and age were comparable in the three groups examined. ApoE e4 was 2.5-fold elevated in AD and 2.0-fold in PD compared to HC although without reaching statistical differences probably due to the small number of subjects of the our cohort.

**Table 2:** Demographic, clinical and genetic characteristics of the individuals enrolled in the study

	Alzheimer’s disease patients (AD)	Parkinson disease patients (PD)	Healthy controls (HC)
N	10	10	10
Gender (M:F)	5:5	7:3	4:6
Age (years)	78.50 ± 5.44	80.22 ± 5.21	78.90 ± 5.95
MMSE	19.07 ± 2.51	–	30
Y&H	–	2.72 ± 0.94	–
*ApoE* ε-4 carriers (%)	50	40	20

Data are expressed as mean ± standard deviation

MMSE: Mini-Mental State Examination

Y&H: Hoehn and Yahr Scale

### HSV-1 viral load and gene expression

HSV-1 viral load in supernatants 24 h post-infection was comparable among the three groups, with the highest values being observed in PD patients (AD: 4.83 × 10^6^ ± 1.98 × 10^6^ copies/ml; PD: 5.31 × 10^6^ ± 1.57 × 10^6^ copies/ml; HC: 5.16 × 10^6^ ± 1.94 × 10^6^ copies/ml). No significant differences were observed in the HSV-1 seronegative group (see Additional file 2).

**Fig. 1:**
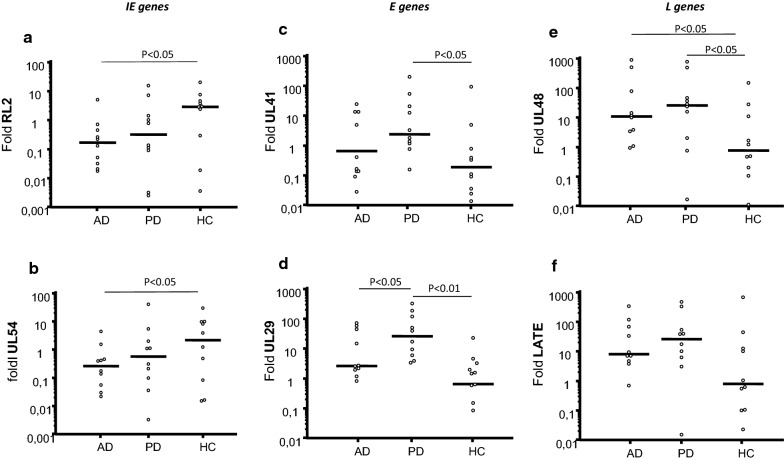
HSV-1 genes expression: Left panels. HSV-1 RL2 (**a**) and UL54 (**b**) immediate early (IE) genes. Central panels. HSV-1 UL41 (**c**) and UL29 (**d**) early (E) genes. Right panels. HSV-1 UL48 (**e**) and LATE (**f**) late (L) genes. HSV-1-infected PBMC of AD and PD patients and Healthy Control (HC) were used to generate the results. Data are expressed as median; statistical significance is shown

The expression of immediate early, early and late HSV-1 genes was analyzed next. Results showed that expression of immediate early genes ICP0 and UL54 was decreased in AD and PD compared to HC; these differences reached statistical significance in the case of AD cells (RL2: p = 0.02 vs. HC. UL54: p = 0.04 vs. HC) (Fig. 1).

The pattern of expression of early and late genes was totally different. Thus, all the four analyzed early and late viral genes were over-expressed upon HSV-1 infection of cells of AD and PD patients compared to HC. In particular, in PD, the expression of the UL41 (p = 0.02) and UL29 (p = 0.003) early genes as well as that of the UL48 (p = 0.04) late gene was significantly increased compared to HC. In AD, expression of the UL48 late gene was significantly increased in comparison with HC (p = 0.04). Interestingly, expression of all early and late genes was higher in PD compared to AD patients, reaching statistical significance for the UL29 early gene (p = 0.04) (Fig. 1). Unormalized qPCR data were summarized in additional file (see Additional file 3). A similar pattern of gene expression observed in HSV-1—uninfected individuals.

### IFN-λ mRNA expression and serum concentration

**Fig. 2:**
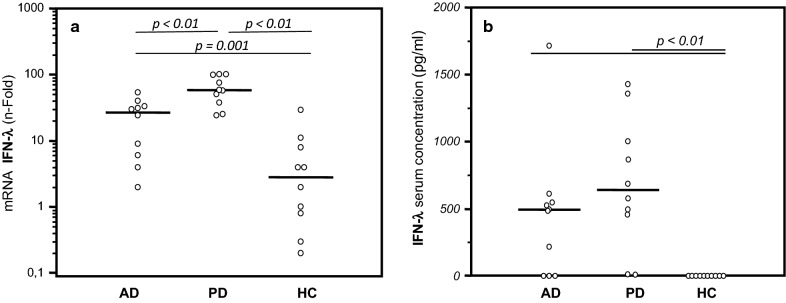
IFN-λ mRNA-expression and serum concentration. **a** *IFN*-λ mRNA expression in HSV-1-infected cells of HSV-1-infected PBMC from AD and PD patients and Healthy Control (HC). **b** IFN-λ serum concentration in AD and PD patients and Healthy Control (HC). Data are expressed as median, each dot represents *IFN*-λ gene or protein production by single individual. Statistical significance is shown

IFN-λ (IL-29) is a cytokine that is part of the innate immune response and is endowed with a potent antiviral activity. IFN-λ (IL-29) mRNA was measured in PBMC that were infected or not with HSV-1,as well as serum concentration was detected in all subjects enrolled. Results showed that IFN-λ mRNA expression was significantly increased in PD and AD patients compared to HC (p = 0.001 and p = 0.003, respectively), and in AD compared to PD (p = 0.005) (Fig. 2). Similar results were obtained when serum concentration of IFN-λ (IL-29) was measured. Thus, IFN-λ (IL-29) serum concentration was significantly increased in AD and PD patients compared to HC (p < 0.01 in both cases) (Fig. 2). When IFN-λ production was measured in supernatant by unstimulated and by HSV-1 stimulated PBMC, this cytokine could not be detected in any sample, possibly because of the low sensitivity of the method used (data not shown). A similar pattern of IFN-λ mRNA expression and serum concentration was observed in HSV-1–uninfected individuals. These results indicate that a much more prompt and potent IFN-λ (IL-29)-mediated antiviral innate immune response is elicited in AD and PD compared to what is observed in HC.

### IL-1β and IL-10 production

Cytokines production was evaluated by immunoassay (ELISA) in supernatants of PBMC that were either cultured in medium alone or infected with HSV-1. Results showed that IL-1β production was already increased in unstimulated cells of AD and PD patients compared to HC, even if these differences were not statistically significant.

**Fig. 3:**
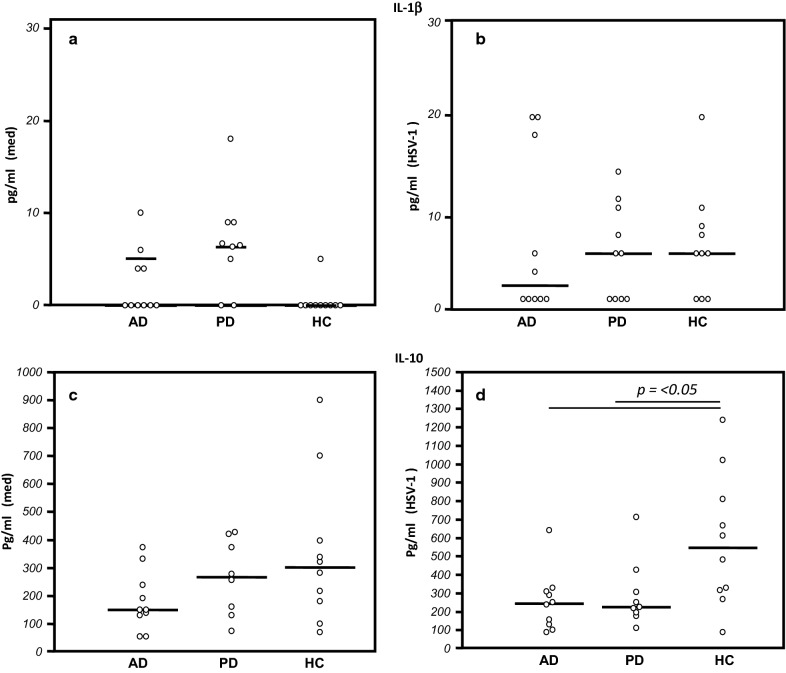
Cytokines production: IL-1β (upper panels) and IL-10 (lower panels) production by PBMC of AD and PD patients and Healthy Control (HC) that were cultured in medium alone (med) (**a**, **c**) or were stimulated with HSV-1 (**b**, **d**). Data are expressed as median, each dot represents IL-1β or IL-10 protein production by single individual. Statistical significance is shown

HSV-1 stimulation induced IL-1β production by cells of HC but did not modify the production of this cytokine by cells of AD and PD. IL-10 production, on the other hand, was slightly increased in HC compared to AD and PD in unstimulated conditions and was significantly increased by HSV-1 infection in HC alone (AD and PD vs. HC p < 0.05) (Fig. 3).

Finally, possibly because of the small number of individuals examined, no correlation was found between the examined immunological and virological parameters and either ApoE or IFN-λ genotypes in any of the groups enrolled in the study. Again similar pattern of cytokine production was observed in HSV-1–uninfected individuals.

## Discussion

Viral infections, and in particular infection with HSV-1, have long been suspected to play a pathogenic role in AD; more recently a possible role for this virus has also been suggested in the pathogenesis of PD. HSV-1 infection remains latent in the nervous system after primary infection;

the hypothesis is supported by research data implicating brain infections by HSV-1 [16, 30]. Viruses are prevalent in AD brains and can evade the host immune system forming latent or chronic infections, interact with genetic and environmental factors to initiate accumulation and/or formation of Aβ, hyperphosphorylation of tau proteins, and inflammation in the brain [21].

Periodic reactivations are hypothesized to be associated with AD and PD, as suggested by a number of results. Thus, in AD: (1) HSV-1 encephalitis involves the hippocampus as well as the temporal and frontal lobes, these are the same brain area that are affected in AD; (2) HSV-1 genome is found in the hippocampus and in the temporal and frontal lobes brains of sporadic AD patients; (3) the combination of *ApoE 4* allele, a genetic risk factor for AD, and HSV-1 infection, greatly augments the risk of developing AD; (4) HSV-1 DNA colocalizes with Aβ; (5) the phosphorylation of tau, which is characteristically seen in AD, is enhanced by HSV-1 infection in in vitro and in animal models [16, 19, 20, 56, 57]; and (6) Aβ and HSV-1 cross-reactive antobodies can be observed in patients [19, 58, 59]. In the case of PD, a possible HSV-1 pathogenetic role is suggested by results indicating that cross reactivity between HSV-1 and α-synuclein stimulates autoimmune responses targetting neurons of the substantia nigra [23]. Taken together these results support the hypothesis that periodic HSV-1 reactivation in the CNS could facilitate the onset of AD and PD, possibly as an effect of direct neuronal damage and of the neuroinflammatory milieu which is driven by viral reactivations [5, 19, 20, 37, 59–61].

Herein we report results obtained in an in vitro system in which peripheral blood immune cells of AD and PD patients as well as of HC were infected with HSV-1. All the individuals enrolled in the study were HSV-1 seropositive, thus, in this in vitro model we evaluated virus-specific secondary immune responses. Results showed that both HSV-1 gene expression and HSV-1-stimulated immune responses are different in patients compared to controls. Thus, the expression of IE (*ICP0* and *ICP27*) genes was reduced in AD and PD, compared to controls. IE genes products activate the transcription of early genes, which are expressed prior to DNA replication, and, subsequently, that of late genes. In contrast to what was observed with IE genes, the expression of both early (*UL41, UL29*) and late (*UL48, LAT*) genes was higher in both groups of patients compared to HC.

Viral replication is initially contained by innate immunity; IFN-λ plays a pivotal role in this process. Recent results showing that IFN-λ production is significantly augmented in patients with a diagnosis of Mild Cognitive Impairment who do not convert into AD over a 24 months period reinforce the idea that that innate immunity and IFN-λ in particular, are important players in protection against AD [8]. The observation herein upon IFN-λ mRNA and serum concentration were significantly increased in both groups of patients suggest that the initial reduction of IE genes transcription is an immune-mediated attempt to obstacle HSV-1 replication, an attempt that is clearly ineffective, as indicated by the augmented transcription rates of E and L viral genes.

Because HSV-1 reactivations is tightly linked to neuroinflammation we analyzed pro- and anti-inflammatory cytokines in our system as well. Results indicated that unstimulated IL-1β production was slightly increased in AD and PD compared to HC whereas that of the anti-inflammatory cytokine IL-10 was reduced in patients compared to controls. Notably, IL-10 production upon in vitro HSV-1 infection was significantly increased in HC alone. IL-10 reduces antigen-stimulation proliferation and dampens immune responses by down modulating the generation of pro-inflammatory cytokines [62–64]. That IL-10 production is reduced in chronic neurodegenerative diseases is known [63, 65]. The results obtained in this in vitro infection system reinforce the observation that inflammation characterizes AD and PD, suggesting that the ability of immune cells of these individuals to secrete IL-10 in response to a viral insult is defective or exhausted. A small group of HSV-1 seronegative AD and HC was also included in the study and interestingly after in vitro infection, immunological and virological results obtained in these individuals overlapped those obtained in HSV-seropositive subjects.

Overall, these results confirm previously published data [8, 65, 66] and support the idea that a constitutive proinflammatory milieu is present in chronic neurodegenerative diseases, including AD and PD.

Notably, ApoE was shown to play a role in the modulation of innate immunity. In humans there are three well-described major isoforms of ApoE: ApoE-ε4, ApoE-ε3 and ApoE-ε2, which are encoded by allelic variants of the *ApoE* gene on chromosome 19. *ApoE*-*ε4* was observed to be more frequent in diseases in which innate immunity plays a role, including AD, and, more recently, viral infection [67, 68]. The presence of single copy apoε4 was also shown to associate with increased production of IL-1β, IL-6, and IFN-λ by TLR-2 and TLR-4 agonists-stimulated cells [69]. Possibly because of the limited number of individuals analyzed in this study, no association between *ApoE* genotype and immunological data was observed; further investigations in larger cohorts of subjects will be needed to verify possible associations between ApoE isofoms and the virological and immunological parameters examined herein. Moreover, although virus-induced pathology is modulated by *ApoE*-*ε4*, it has been reported that ApoE does not modulate HSV shedding [70] so that similar viral loads are to be expected independently of ApoE genotype.

Herein, because IL-1β is also endowed with direct antiviral properties it is nevertheless tempting to speculate that a constitutive production of this cytokine by cells of AD and PD patients could be an additional attempt to prevent viral reactivation.

## Conclusion

Although further studies will be necessary to confirm these findings, results herein indicate that, at least in our in vitro system, HSV-1-specific immune responses differ when healthy controls are compared to AD and PD. In these patients such responses could be geared toward a ineffective control of viral replication through innate immunity-mediated mechanisms.

## Supplementary information

**Additional file 1.** Experiment performed in THP-1 cell line to determine the best timing of HSV-1 gene expression.

**Additional file 2.** Summary statistics table of HSV-1 genes and IFN-lambda mRNA expression, and cytokines concentration.

**Additional file 3.** Raw data of HSV-1 gene expression.

## Data Availability

The data that support the findings of this study are available on request from the corresponding author [FLR]. The dataset supporting the conclusions of this article is included within the article and its additional file.

## Abbreviations

Aβ: amyloid-beta
AD: Alzheimer’s disease
α-syn: alpha-synuclein
ANOVA: analysis of variance
BBB: blood brain barrier
CNS: central nervous system
CDR: Clinical Dementia Rating Scale
DMEM: Dulbecco’s modified Eagle medium
E: early genes
FBS: fetal bovine serum
HC: healthy control
HSV-1: herpes simplex virus type 1
IFN-λ: IFN-lambda
IE: immediate early
IL-1β: Interleukin-beta
IL-10: interleukin-10
L: late genes
MMSE: MiniMental State Examination
MOI: multiplicity of infection
MRI: magnetic resonance imaging
PBMC: peripheral blood mononuclear cells
PD: Parkinson’s disease
