# Supplementary material for: Herpes simplex virus-1 (HSV-1) infection induces a potent but ineffective IFN-λ production in immune cells of AD and PD patients
Source: J Transl Med. 2019 Aug 27;17:286. doi: 10.1186/s12967-019-2034-9 (PMC6712644; doi:10.1186/s12967-019-2034-9)
Supplement: Supplementary file 1 — Additional file 1. Experiment performed in THP-1 cell line to determine the best timing of HSV-1 gene expression. [file 12967_2019_2034_MOESM1_ESM.pdf]

Additional file

To determine the best timing of expression of viral genes, parallel experiments in human monocytic cell line THP-1, obtained from IZSLER (Istituto Zooprofilattico Sperimentale della Lombardia e Dell'Emilia Romagna, IT) and grown in RPMI 1640 supplemented with 10% FBS, 2mM L-glutamine, and 1% penicillin (medium) (Invitrogen Ltd, Paisley, UK) were conducted. $1,3*10^6$ cells for well were plated in 96-wells plate and were incubated at 37°C in 5% CO_2 with/without HSV-1 1MOI for 1 h in serum-free RPMI; after infection, washed with phosphate-buffered saline (PBS), cells were cultured in 12 wells plate with RPMI 1640 supplemented with 2mM L- glutamine, 1% penicillin and 2% FBS for 1, 4, 6, 12, 24 hours. On the bases of these experiments, we decided to analyze the immediate early genes 1 hours p.i, the early genes 4 hours p.i and late genes 6 hours p.i.
