# Supplementary material for: Herpes simplex virus-1 (HSV-1) infection induces a potent but ineffective IFN-λ production in immune cells of AD and PD patients
Source: J Transl Med. 2019 Aug 27;17:286. doi: 10.1186/s12967-019-2034-9 (PMC6712644; doi:10.1186/s12967-019-2034-9)
Supplement: Supplementary file 2 — Additional file 2. Summary statistics table of HSV-1 genes and IFN-lambda mRNA expression, and cytokines concentration. [file 12967_2019_2034_MOESM2_ESM.pdf]

	HSV-1 seronegative AD	HSV-1 seronegative HC
HSV-1 viral load (copies/ml)	$1.13x10^6$±0.58 $x10^6$	$2.30x10^6$±0.64 $x10^6$
Fold ICP0	0.09±0.01	4.92±0.23**
Fold UL54	0.28±0.02	2.00±0.03**
Fold UL41	1.94±0.18	0.84±0.15**
Fold UL29	1.77±0.23	0.91±0.18*
Fold UL48	5.93±0.21	0.92±0.05**
Fold LAT	6.44±0.25	0.70±0.02**
IFN-λ (pg/ml)	400±123.23	0.02±0.001*
IFN-λ mRNA (Fold-change/medium)	0.17±0.025	0.02±0.001**
IL-10 (med) (pg/ml)	169.53±98.23	369.70±102.3
IL-10 (HSV-1) (pg/ml)	225.74±105.35	565.94±203.21
IL-1β (med) (pg/ml)	2.21±2.80	0.50±1.56
IL-1β (HSV-1) (pg/ml)	8.60±1.98	5.60±4.9

Summary statistics table of: HSV-1 (IE, E and L) genes fold, IFN-λ mRNA fold quantified by qPCR; cytokines IL-10, IL-1β concentration (pg/ml) in supernatants of cultured PBMC and IFN-λ seum concentration evaluated by ELISA. Data were shown as means ± SD. Statistical analyses between HSV-1 seronegative AD *versus* HC were shown. * pvalue ≤ 0.005; **pvalue ≤ 0.001.
