# Supplementary material for: Herpes simplex virus-1 (HSV-1) infection induces a potent but ineffective IFN-λ production in immune cells of AD and PD patients
Source: J Transl Med. 2019 Aug 27;17:286. doi: 10.1186/s12967-019-2034-9 (PMC6712644; doi:10.1186/s12967-019-2034-9)
Supplement: Supplementary file 3 — Additional file 3. Raw data of HSV-1 gene expression. [file 12967_2019_2034_MOESM3_ESM.pdf]

Summary of unnormalized data.

			HSV-1 gene expression (cycle threshold)									HSV-1 gene expression (Fold)					
ID	AbHSV-1	PATOLOGY	YWHAZ 1h p.i.	ICP0	ICP27	YWHAZ 4h p.i.	ICP8	UL41	YWHAZ 6h p.i.	VP16	LAT	ICP0	ICP27	ICP8	UL41	VP16	LAT
17R129	POS	PD	25,98	28,33	24,47	28,45	24,59	29,21	28,45	23,77	21,2	0,24	0,27	5,78	4,11	2,25	5,62
17R165	POS	PD	22,7	30,53	27,2	23,12	21,92	25,95	23,12	24,14	22,21	0,00	0,00	3,72	2,93	1,26	2,73
17R187	POS	PD	31,56	26,78	22,45	29,85	22,32	26,31	29,85	27,76	21,73	15,56	40,22	299,00	242,19	10,84	404,50
17R188	POS	PD	25,88	25,03	21,95	26,9	22,03	25,95	26,9	24,97	22,35	1,02	1,11	47,31	40,22	9,70	34,06
17R285	POS	PD	21,55	29,04	22,58	24,68	23,67	29,32	24,68	23,1	22,04	0,00	0,04	3,26	0,84	7,61	9,06
17R287	POS	PD	26,43	25,13	21,68	26,4	23,08	27,57	26,4	30,68	33,06	1,39	1,96	16,16	9,25	0,13	0,01
17R289	POS	PD	27,4	26,96	23,5	29,19	23,1	27,16	29,19	24,26	21,58	0,77	1,09	110,20	85,04	77,60	284,05
17R299	POS	PD	27,48	23,82	21,26	26,12	23,64	27,97	26,12	23,61	22,71	7,16	5,43	9,03	5,78	14,50	15,45
17R189	POS	PD	26,41	29,06	26	29,89	25,36	32,05	28	25,69	23,43	0,09	0,10	37,37	4,66	12,62	34,54
17R325	POS	PD	26,3	28,36	24,24	30,47	23,72	30,1	28,28	23,75	23,25	0,14	0,30	174,13	26,91	58,81	47,50
16R324	POS	AD	24,33	24,71	22,03	23,47	23,08	29	29,09	24,04	21,45	0,44	0,36	2,12	0,45	84,33	290,02
15R221	POS	AD	24,31	26,47	23,58	22,86	22,24	27,44	25	22,04	20,65	0,13	0,12	2,49	0,87	19,81	29,65
15R44	POS	AD	25,82	27,25	23,59	26,45	25,74	31,72	29,57	25,45	23,72	0,21	0,34	2,65	0,54	3,29	6,23
15R155	POS	AD	24,61	28,79	26,51	22,16	22,67	27,42	22,61	23,32	23,82	0,03	0,02	1,14	0,54	1,56	0,63
14R342	POS	AD	25,95	29,46	27,42	26,51	26,29	30,21	29,29	24,73	23,16	0,05	0,03	1,88	1,60	60,05	101,83
15R45	POS	AD	28,89	28,63	23,22	28,89	23,51	27,77	28,89	27,38	26,37	0,68	3,71	67,37	45,25	7,25	8,34
17R146	POS	AD	26,04	22,99	21,88	27,52	22,46	27,07	28,32	29,16	22,97	4,69	1,30	53,97	28,44	1,42	59,30
17R238	POS	AD	27,92	29,1	25,5	26,51	21,81	26,06	24,8	24,53	23,26	0,25	0,39	42,05	28,44	3,07	4,23
17R250	POS	AD	24,8	29,84	25,38	23,26	24,28	29,86	24,8	23,45	22,7	0,02	0,05	0,80	0,21	6,49	6,23
17R266	POS	AD	25,21	30,01	24,16	24,76	21,63	25,46	25,21	24,02	23,97	0,02	0,15	14,16	12,82	5,81	3,43
17R93	POS	HC	27,34	25,27	23,26	26,47	24,94	30,27	27,14	31,77	33,24	2,38	1,23	4,67	1,49	0,10	0,02
17R47	POS	HC	25,66	26,63	25,49	24,66	28,93	29,94	29,94	30,56	31,33	0,29	0,08	0,08	0,54	1,66	0,55
17R43	POS	HC	24,12	29,04	26,3	28,22	31,66	25,57	26,01	28,71	26,62	0,02	0,02	0,15	130,69	0,39	0,95
17R42	POS	HC	27	24,27	24,26	21,91	23,39	28,67	28,42	30,56	32,34	3,76	0,49	0,58	0,19	0,58	0,10
17R270	POS	HC	28,09	25,07	21,02	23,02	23,2	29,36	24,38	24,73	25,92	4,59	9,78	1,43	0,26	2,00	0,50
17R269	POS	HC	28	25,67	22,28	25,75	22,01	26,46	28,41	27,13	25,79	2,85	3,84	21,62	12,73	6,18	8,94
17R268	POS	HC	26,95	21,81	18,3	23,03	23,11	28,07	27,09	25,01	24,15	19,97	29,24	1,53	0,63	10,76	11,16
17R259	POS	HC	29,94	27,55	22,91	24,58	23,13	28,19	24,75	26,19	16,1	2,97	9,51	4,42	1,71	0,94	584,07
17R263	POS	HC	29,26	25,53	22,47	21,78	23,16	29,2	23,33	24,71	27,31	7,52	8,06	0,62	0,12	0,98	0,09
14R105	POS	HC	27,49	27,8	24,1	25,23	23,9	28,03	29,2	25,69	24,44	0,46	0,76	4,07	2,99	29,00	39,397
